# Intranasal calcitonin gene-related peptide administration impairs fear memory retention in mice through the PKD/p-HDAC5/Npas4 pathway

**DOI:** 10.1038/s41598-022-05518-y

**Published:** 2022-01-27

**Authors:** Narumi Hashikawa-Hobara, Yoshikazu Yoneyama, Kyoushiro Fujiwara, Naoya Hashikawa

**Affiliations:** Department of Life Science, Okayama University of Science, 1-1 Ridai-cho, Kita-ku, Okayama, 700-0005 Japan

**Keywords:** Fear conditioning, Blood-brain barrier

## Abstract

The calcitonin gene-related peptide (CGRP) suppresses fear memory retention in mice. Although intracerebroventricular administration of CGRP alters the fear memory processes, making it a promising therapeutic strategy for post-traumatic stress disorder (PTSD), direct brain injection into patients is not practical. Therefore, we propose that intranasal application may be an effective way to deliver CGRP to the brain. This study tested whether CGRP nasal administration exerts the same effect as intracerebroventricular administration using C57BL6J mice. The amount of CGRP in the cerebrospinal fluid and hippocampus 30 min after nasal administration of CGRP was significantly higher when compared with saline. Intranasal CGRP also elicited photophobic behaviors similar to intracerebroventricular injection. Moreover, intranasal CGRP decreased fear memory retention but did not affect reactivation and extinction of fear memory. We found intranasal CGRP significantly increased the expression of protein kinase D (PKD), phosphorylated histone deacetylase 5 (p-HDAC5) and neuronal PAS domain protein 4 (Npas4) in the hippocampus. CGRP-mediated impairment of fear memory and Npas4 expression increases were attenuated significantly by the CGRP receptor antagonist BIBN4096. Together, our data demonstrate that intranasal CGRP delivery activates the PKD/p-HDAC5/Npas4 pathway, decreases fear memory retention.

## Introduction

Post-traumatic stress disorder (PTSD) is a disorder of learning and memory caused by exposure to frightening or life-threatening events. The lifetime prevalence of PTSD in adults is approximately 8% in the United States^1^. There are few medication options for the treatment of PTSD, and the selective serotonin reuptake inhibitors have been used as the first-line pharmacological treatment. However, the response rate of this drug is less than 20–30%^2^. Therefore, the development of new pharmacological strategies for PTSD is required. Our previous study showed that calcitonin gene-related peptide (CGRP) is involved in suppressing fear memory retention^3^. CGRP is a 37-amino acid neuropeptide released from capsaicin-sensitive sensory nerves^4^. CGRP is widely distributed in both the central and peripheral nervous systems^5^. CGRP is a potent vasodilator with some beneficial cardio-protective functions. It has also been shown to play a role in limiting adverse inflammatory events, modulating pain levels, and acting as an anti-depressant^6–9^.

As CGRP cannot cross the blood–brain barrier (BBB) due to its molecular size, CGRP has been directly injected into the brain of rodents for central nervous system experiments. In contrast, there are a few experimental reports that suggest that the noninvasive, intranasal (i.n.) administration of CGRP is more effective than intravenous delivery^10,11^. Drug delivery to the brain via the nasal mucosa is a rapidly developing field that holds great promise, especially for anti-Alzheimer disease agents^12^. Additionally, in clinical trials, i.n. administration of insulin did not increase circulating levels of insulin^13^, indicating that the nose-to-brain pathway is able to preferentially deliver peptides to the brain. Our previous studies have reported that intracerebroventricular (i.c.v.) administration of CGRP immediately after fear memory exposure impairs fear memory retention in C57BL6J mice^3^. Therefore, we propose that i.n. delivery of CGRP may be a more appropriate and feasible strategy for treating PTSD.

In this study, we assessed whether i.n. CGRP administration to mice would modulate fear memory processing. Furthermore, we focused on CGRP signaling via the protein kinase D (PKD)/phosphorylated histone deacetylase 5 (p-HDAC5)/neuronal PAS domain protein 4 (Npas4) pathway in the mouse hippocampus. In this study, we found that i.n. CGRP application suppresses fear memory retention and increases Npas4 expression via CGRP receptor activation. Together, our data indicate that i.n. CGRP administration could be a viable potential alternative to direct brain injection for delivering CGRP to PTSD patients.

## Results

### Intranasal administration delivers CGRP to cerebrospinal fluid and the hippocampus

**Figure 1:**
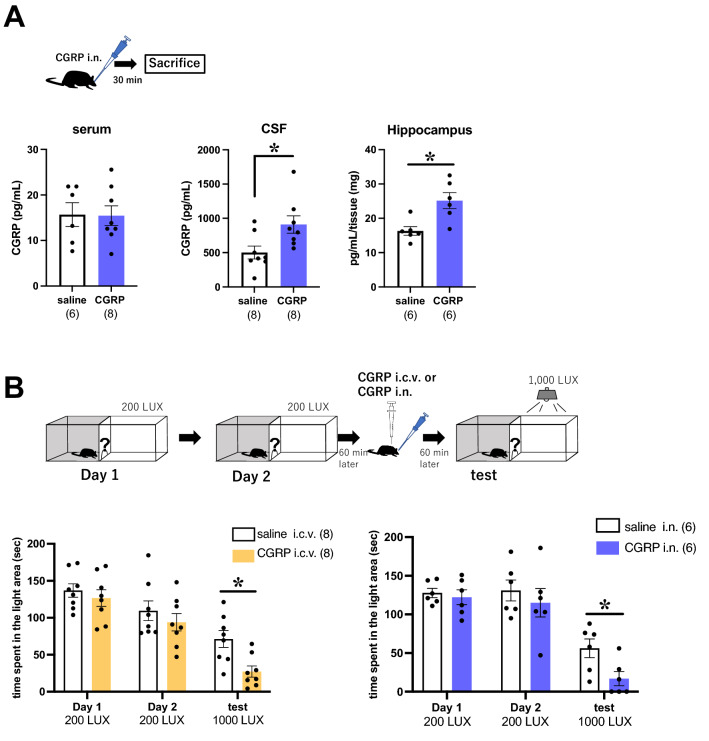
Changes in CGRP distribution and photophobic behavior after intranasal administration of CGRP. (**A**) CGRP expression was assessed in the serum, cerebrospinal fluid (CSF) and hippocampus using ELISA 30 min after CGRP application. (**B**) Photophobic behavioral assay results in saline and CGRP-treated mice. Each bar indicates the mean ± S.E.M. *p < 0.05, Welch’s *t* test. Numbers in parentheses indicate the number of animals in each group.

We first determined whether i.n. administration of CGRP had enough delivery efficiency to enter the cerebrospinal fluid (CSF) or hippocampus in mice. Thirty minutes after i.n. administration of CGRP, serum levels of CGRP were not increased (Fig. 1A, saline (n = 6), CGRP (n = 8)). In contrast, CSF levels of CGRP were significantly higher than those observed for i.n. saline administration (Fig. 1A, Welch’s t test, p = 0.0225, saline (n = 8), CGRP (n = 8)). These experiments demonstrate that delivered CGRP is present in the CSF 30 min after i.n. administration. We therefore similarly examined CGRP levels in the hippocampus and found significantly higher levels than that observed in saline-treated animals (Fig. 1A, Welch’s t test, p = 0.0291, saline (n = 6), CGRP (n = 6)). Collectively, these data indicate that i.n. administration delivers CGRP to the CSF and hippocampus in mice.

### Intranasal administration of CGRP elicits known physiological behaviors

I.c.v. administration of CGRP has been shown to cause a photophobic behavioral response in mice^14^. We next sought to determine whether i.n. CGRP elicits a similar response by using the photophobia test according to the protocol from Recober et al.^14^. Mice were habituated to the apparatus and allowed to freely explore both the light (200 LUX) and dark compartments of the box for 2 days. The residence times in the light compartment in these sessions were approximately 130 s in both i.n. and i.c.v. administered CGRP. We then measured the time spent in a 1000 LUX light box 60 min after CGRP treatments. We found the residence times were significantly reduced by 38% and 30% with i.c.v. and i.n. administration, respectively, (Fig. 1B, Welch’s t test, i.c.v. p = 0.0072, saline (n = 8), CGRP (n = 8), i.n. p = 0.0284, saline (n = 6), CGRP (n = 6)). These results indicate that i.n. application of CGRP revealed photophobic behavior similar to that seen with direct brain injection.

### Effects of intranasal administration of CGRP on contextual fear memory

**Figure 2:**
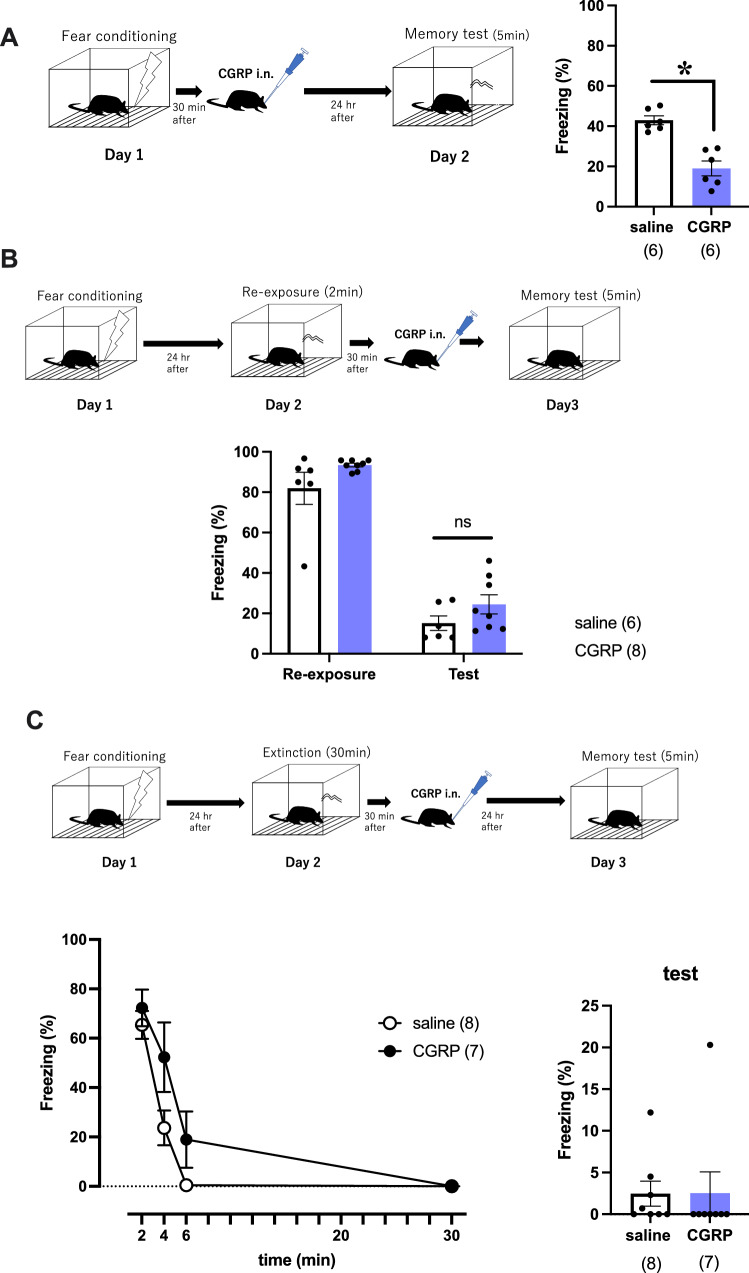
Effects of intranasal CGRP on contextual fear memory. (**A**) To study the effect of CGRP on memory retention of contextual fear, CGRP was nasally administered 30 min after fear conditioning, and freezing behavior was observed 24 h later. (**B**) To investigate the effect of CGRP on the reconsolidation of contextual fear memory, mice were fear conditioned and re-exposed 24 h later for 2 min. CGRP was administered intranasally 30 min after re-exposure, and freezing behavior was observed on Day 3. (**C**) For fear memory extinction, mice were fear conditioned and 24 h later were placed back in the box for 30 min for extinction of the of contextual fear memory. CGRP was administered intranasally 30 min after the 30-min extinction, and freezing behavior was observed on Day 3. Each bar indicates the mean ± S.E.M. *p < 0.05, Welch’s *t* test. Numbers in parentheses indicate the number of animals in each group.

We have previously reported that CGRP i.c.v. administration reduces fear memory retention^3^. In this study, we investigated whether i.n. administration of CGRP affects contextual fear memory. First, we investigated whether exogenous CGRP affects memory retention of contextual fear. Mice were given i.n. CGRP or saline 30 min after a foot shock and were tested 24 h later. CGRP treatment significantly decreased freezing time (Fig. 2A, Welch’s t test, p = 0.0005, saline (n = 6), CGRP (n = 6)). In order to exclude the factors of hand-holding stress, we also evaluated effect of CGRP i.n. under anesthesia (Supplementary Fig. S1). Anesthesia did not affect CGRP-mediated freezing percentage (20%), which was same as without anesthesia (Fig. 2A). Next, we examined whether CGRP affects the reactivation of contextual fear memory. In our previous data, CGRP i.c.v. administration after re-exposure prolonged freezing time^15^. According to this data, it is expected i.n. administration of CGRP would increase freezing time. CGRP or saline were administered 30 min after a 2-min reactivation period, followed by testing 24 h later. CGRP did not affect freezing time compared with saline treatment (Fig. 2B, saline (n = 6), CGRP (n = 8)). Finally, we examined whether CGRP affects fear memory extinction. Mice received CGRP after re-exposure for 30 min to erase fear memory and after 24 h were subjected to a freezing test. We observed that CGRP did not affect freezing time compared with saline treatment, indicating that CGRP does not have an effect on eliminating fear memory (Fig. 2C, saline (n = 8), CGRP (n = 7)).

### Intranasal CGRP increases PKD, p-HDAC5, and Npas4 levels in the mouse hippocampus during fear conditioning

**Figure 3:**
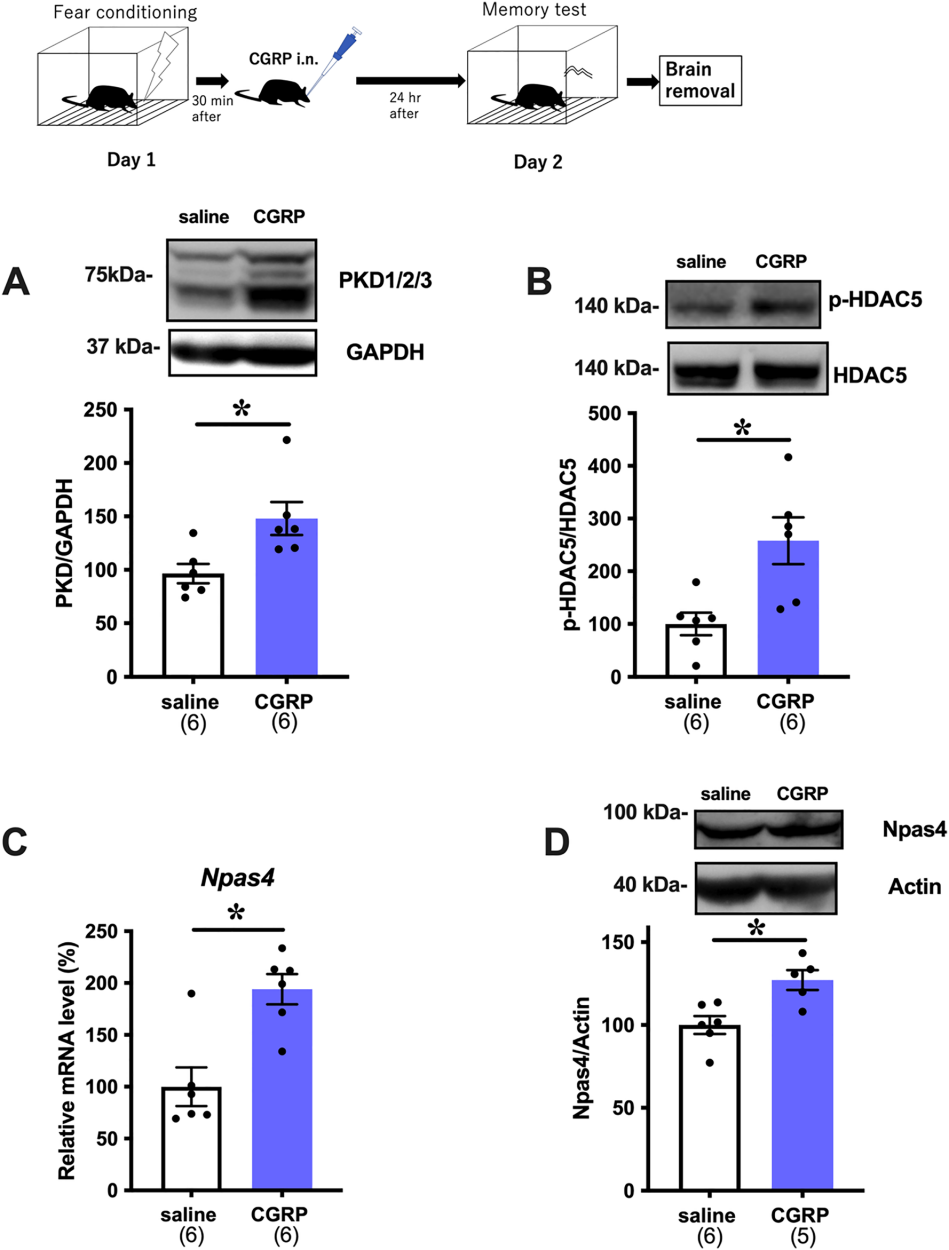
Effects of intranasal administration of CGRP on the PKD/p-HDAC5/Npas4 pathway. (**A**) PKD protein levels in saline and CGRP-treated mice, (**B**) Phosphorylated HDAC5 expression in saline and CGRP-treated mice, (**C**) *Npas4* mRNA levels in saline and CGRP-treated mice, and (**D**) Npas4 protein expression in saline and CGRP-treated mice. Each bar indicates the mean ± S.E.M. *p < 0.05, Welch’s *t* test. Numbers in parentheses indicate the animal numbers for each group.

To investigate the mechanism of i.n. CGRP administration on memory retention deficits, we focused on Npas4, which is a transcription factor that has a well-established role in fear memory processing^16^. A previous report from our group demonstrated that i.c.v. CGRP reduced fear memory retention by increasing Npas4 expression, as well as PKD and p-HDAC5, in the mouse hippocampus^3^ To confirm whether the PKD/p-HDAC5/Npas4 signaling cascade is altered with i.n. administration of CGRP, as it is with i.c.v. administration, we first analyzed PKD levels in the hippocampus of mice 24 h after they were exposed to foot shock. We found that i.n. CGRP significantly increased PKD expression (Fig. 3A, Welch’s t test, p = 0.0206, saline (n = 6), CGRP (n = 6)). Next, we analyzed p-HDAC5 levels. We have previously reported that i.c.v. CGRP increases the phosphorylation of Ser 498 in HDAC5^3^. p-HDAC5 cannot translocate into the nucleus to suppress the transcription of Npas4. Similarly, we found that i.n. CGRP significantly increased p-HDAC5 levels (Fig. 3B Welch’s t test, p = 0.0142, saline (n = 6), CGRP (n = 6)). Congruent with the idea that p-HDAC5 elevates Npas4 expression, *Npas4* mRNA and protein levels were significantly increased by i.n. CGRP. (Fig. 3C Welch’s t test, p = 0.0015, saline (n = 6), CGRP (n = 6), Fig. 3D Welch’s t test, p = 0.0092, saline (n = 6), CGRP (n = 5)).

### CGRP receptor antagonist BIBN4096 inhibits CGRP-mediated memory disability by suppressing Npas4 expression

**Figure 4:**
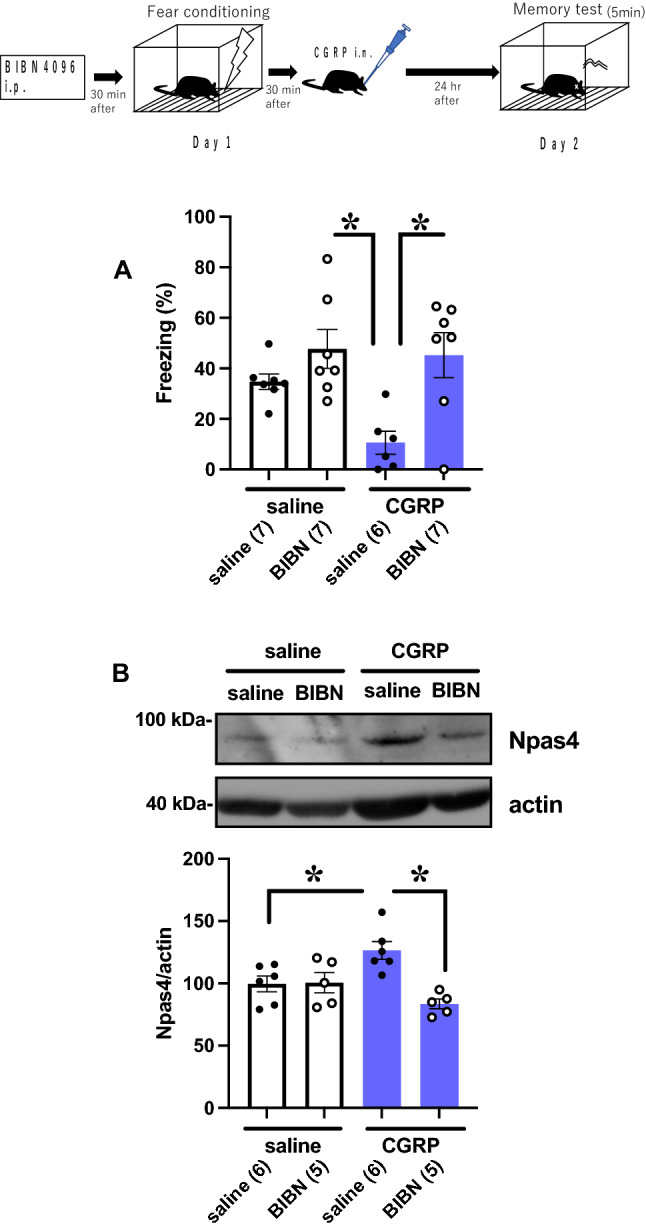
Intranasal CGRP attenuates fear memory retention via the CGRP receptor with an increase in Npas4 expression. (**A**) Effects of CGRP receptor antagonist BIBN4096 (1 mg/kg) on fear memory retention. (**B**) Effects of BIBN4096 on the levels of Npas4 protein expression. Each bar indicates the mean ± S.E.M. *p < 0.05, Tukey–Kramer test. Numbers in parentheses indicate the animal numbers for each group.

As i.n. administration of CGRP activates the PKD/p-HDAC5/Npas4 pathway, we next asked whether CGRP receptor blockade suppress the CGRP-mediated decrease in fear memory retention and Npas4 protein expression. We administered the CGRP receptor antagonist BIBN4096 intraperitoneally and then evaluated freezing behavior after 24 h. We observed that BIBN4096 significantly inhibited CGRP-mediated memory retention impairment (Fig. 4A, BIBN versus CGRP; p = 0.004, CGRP versus BIBN + CGRP; p = 0.0073 with Tukey–Kramer test, saline (n = 7), BIBN (n = 7), CGRP (n = 6), BIBN + CGRP (n = 7)). As expected, in the presence of BIBN4096, CGRP failed to increase Npas4 protein levels (Fig. 4B, saline versus CGRP; p = 0.0364, CGRP versus BIBN + CGRP; p = 0.0013 with Tukey–Kramer test, saline (n = 6), BIBN (n = 5), CGRP (n = 6), BIBN + CGRP (n = 5)). These data demonstrate that CGRP suppresses fear memory retention by increasing Npas4 via the CGRP receptor.

## Discussion

The major finding of this study is that i.n. administration of CGRP can suppress fear memory retention in the same way as i.c.v. administration. Specifically, we show that CGRP increases Npas4 levels via the CGRP receptor, indicating that activation of the PKD/p-HDAC5/Npas4 pathway plays a role in memory retention.

Drug delivery from the nasal mucosa to the brain is a rapidly progressing area in basic and clinical research. It has been reported that drugs administered into the nasal cavity can be directly transferred to the brain via cerebrospinal fluid or can reach the brain directly without inference by the BBB^17,18^. The nasal mucosa is innervated by olfactory and trigeminal nerves providing a direct connection between nose and brain. Therefore, olfactory and trigeminal pathways are direct drug delivery routes, without the need to pass into the systemic circulation and cross the BBB to reach the brain^19^. Clinical trials for treating Alzheimer’s disease with insulin nasal spray^20^ and autism spectrum disorders with oxytocin nasal spray^21^ have reported that small molecule peptides delivered to the central nervous system by i.n. administration improved cognitive function and social behavior, respectively. Oxytocin is a peptide consisting of nine amino acids, and insulin is composed of two peptide chains, A and B, which consist of 21 and 30 amino acids, respectively, linked by two disulfide bonds. Considering that CGRP is a peptide of 37 amino acids, it is highly possible that the same effect as seen with i.c.v. administration could be obtained. In our present study, we observed similar photophobic behaviors with both i.c.v. and i.n. CGRP administration.

CGRP has been reported to have a wide distribution in the central and peripheral nervous systems. CGRP is well known as a key component in migraine-related headache induction^22^. However, CGRP has been reported to show some protective effects on both ischemic brain injury, with increasing levels of vascular endothelial growth factor, which provides protection from neuronal injury^10^, and vasospasm following subarachnoid hemorrhage^23^ due to its potent vasodilation effects. Additionally, CGRP has been shown to exert an anti-inflammatory effect in lipopolysaccharide-induced kidney injury^8^. Moreover, regarding effects on the central nervous system, we have reported that CGRP pre-administration alleviates stress-induced depression-like behavior with elevated nerve growth factor expression^7^. Furthermore, administration of CGRP in extinction training models diminishes fear memory^24^, and we have also demonstrated that i.c.v. administration of CGRP immediately after exposure to fear conditioning decreases fear memory retention^3^. Thus, CGRP has protective effects for cardiovascular injury, kidney injury, and stress behaviors. In the cardiovascular system, it has been reported that nasal administration of CGRP reduces cerebral vasospasm^10^ and cerebral ischemic injury^11^. Nevertheless, the potential behavioral effects of CGRP i.n. administration are still not defined, especially with regard to fear memory. In this study, we found that i.n. CGRP can reach the hippocampus and induce decreases in fear memory retention with a concomitant elevation of Npas4 expression. These data demonstrate that CGRP could be a practical approach to diminish fear memory.

While these results are promising, this study also has some limitations. First, CGRP is well known as a key player in the pathogenesis of migraines^25^. For decades, CGRP-targeted drugs such as the selective CGRP receptor antagonists BIBN4096^26^ or CGRP antibody drugs have been studied for effective treatments for migraine headaches. Recently, the small molecule gepants that block the CGRP receptor and four anti-CGRP monoclonal antibodies, such as erenumab, have received FDA approval for the treatment of acute migraines^27^. Therefore, exogenous CGRP-induced migraine-like effects are unlikely to be avoided. Wattiez et al. have demonstrated that 1 h after intraperitoneal injection of CGRP mouse activity was attenuated and light aversive behaviors were induced^28^. Thus, when considering the clinical application of CGRP i.n. administration, likely headache side effects cannot be ignored. Second, CGRP is a multifunctional neuropeptide that is widely distributed in the central and peripheral nervous systems, indicating that CGRP could have other side effects, such as gastrointestinal issues including diarrhea. Indeed, intraperitoneal injection of CGRP induced diarrhea, which was suppressed by either olcegapant, a CGRP receptor antagonist, or anti-CGRP antibodies in mice^29^. Additionally, CGRP can function in the transmission of nociception^30,31^. In the present study we applied 0.1 mM CGRP in a total fluid amount of 5 μL (approximately 20 nmol/kg) into each nostril using a manual micropipette. Encouragingly, mice that received i.n. CGRP did not apparently experience itching, severe discomfort, or any inflammatory responses. As CGRP is released from peripheral sensory nerves by a pain stimulus rather than acting as an algogenic substance, it is thought that CGRP does not directly induce a pain reaction. There has also been an investigation looking at whether CGRP affects taste response. Huang et al. have reported that CGRP acting on type III^32^ sour and salt taste cells^33^ via the CGRP receptor increases serotonin release and suppresses ATP release from type II cells^34^ (sweet, umami, bitter)^32–34^. Based on these previous reports, CGRP could have a sour or salty taste. Altogether, CGRP has wide-ranging functions and may have adverse effects that were not detected in the present study, therefore, further study into this important area is needed.

In this study, we focused on PKD/pHDAC5/Npas4 signaling, which has previously been reported to play a role in inhibiting fear memory in mice hippocampus^3^. Here we demonstrate that the CGRP receptor antagonist BIBN4096 suppresses CGRP-mediated Npas4 increases and inhibits the suppression of fear memory retention. We further show that Npas4 increases in the hippocampus are a result of the signaling pathway from CGRP. It is well known that CGRP activates protein kinase A as a second messenger by CGRP receptor activation^35^. More recently, CGRP has been shown to regulate both protein kinase C and MAP kinase^36–38^. In a previous study, we have reported that CGRP also activates PKD and that action of CGRP was inhibited by PKD knockdown^3^. PKD is a serine/threonine kinase that has been implicated in the regulation of the phosphorylation of HDAC5 at Ser 498^39,40^. Consistent with previous reports, and similar to i.c.v. administration, we observed that i.n. CGRP increases PKD expression. Even though a previous study confirmed that PKD inhibition suppress the effects of CGRP, this present study did not conduct a PKD inhibition experiment. However, we believe that similar results would be obtained. There are still few reports on CGRP activation of PKD, and further study is required.

In summary, we report that i.n. CGRP is effective in reducing fear memory retention via the PKD/p-HDAC5/Npas4 pathway. As CGRP is effective immediately after fear exposure, a simple i.n. administration may be appropriate in clinical settings. Taken together, these results suggest that i.n. application of CGRP could be a practical approach to diminish fear memory.

## Methods

All methods were performed in accordance with the relevant guidelines and regulations.

### Animals

Six-week-old C57BL/6 J male mice were purchased from Japan SLC, Inc. (Shizuoka, Japan). Mice housed in the Animal Research Center of Okayama University of Science were controlled with an ambient temperature of 22 °C, with 50 ± 10% relative humidity, and a 12-h light/dark cycle (lights on at 7:00 AM). Mice were housed in groups of 5 to 6 per cage (width 235 × depth 353 × height 160 mm) with wood flake litter for 2 weeks and then used in experiments starting at 8 weeks of age. All animal procedures were approved by the ethics committee of the Animal institutional Research Center of Okayama University of Science (authorization numbers 2021002), in accordance with the ARRIVE guidelines to use the minimum number of mice necessary. A total of 123 mice were used with 16 mice being used for ELISA assays, 28 mice used in photophobic behavioral paradigms, and 79 mice used for fear conditioning tests and mRNA or protein analyses. As the behavioral tests showed variations in mice, each group was set to around eight mice. The biochemical parameters were around six animals per group. Mice were randomly divided into saline, i.n. CGRP, or i.c.v. CGRP groups.

### Drug treatments

Rat CGRP (PEPTIDE Institute, Inc. Osaka, Japan) and the CGRP antagonist BIBN4096 (Sigma Aldrich CO. LLC. Japan) were diluted in saline and dimethyl sulfoxide, respectively. Isoflurane (1.5%–2.0%) was used for brief anesthesia during i.c.v. injections. Drug administration was performed by direct injection into the right lateral ventricle, through the intact scalp, aiming at 1 mm posterior to the bregma and 1 mm to the right of the midline, as described previously^3,7^. For i.n. CGRP delivery, mice were hand-restrained, placed in a supine position, and given 5.0 μL CGRP (0.1 mM) or saline delivered into the nares using a standard pipette. The CGRP solution was applied in small drops alternately in each nostril and was actively inhaled. BIBN4096 (1 mg/kg) was administered by intraperitoneal injection, 30 min before fear conditioning.

### Behavioral assessments

#### Photophobia test

We modified a light/dark box apparatus (30 cm × 20 cm × 20 cm, width × length × height) to implement a photophobia test according to Recober et al.^14^. The box contained a partition between the light and dark compartments, with an entrance (5 cm × 5 cm) that allowed for free movement. Mice were always placed at the joint between light-facing boxes facing the light and allowed to freely move between the light and dark compartments for 5 min. In the baseline behavioral test, light intensity was regulated at approximately 200 LUX. All behaviors were recorded and measured as time spent in the light area. Two days after baseline testing in the light avoidance assay, each mouse was treated with saline or CGRP by i.c.v. injection or i.n. administration. After 60 min, the light intensity was increased to 1000 LUX, and time spent in the light area was measured again. After the test or training, mice were returned to their home cages. The floor of the apparatus was cleaned with 70% ethanol after testing.

#### Contextual fear conditioning experiments

Contextual fear conditioning paradigms were performed as previously described^3,41^. To examine the effects of i.n. CGRP on memory retention, mice were placed in a round chamber apparatus (diameter: 16.5 cm; height: 7.5 cm). After 2 min of habituation in the chamber, mice were given foot shocks (0.3 mA, four times at 2-s intervals). Mice were then given i.c.v. CGRP, i.n. CGRP, or saline 1 h after receiving the foot shocks. The following day, freezing behavior was observed in the same chamber, in which mice were given foot shocks for 5 min. Chambers were cleaned using 70% ethanol between each trial.

To examine the effects of i.n. CGRP on fear memory reactivation, mice were fear conditioned in a chamber with foot shocks and returned to their home cages. The following day (Day 2), mice were placed in the same chamber but without foot shocks to reactivate the fear memory, followed by administration of CGRP or saline by nasal administration. On the following day (Day 3), the freezing behavior was observed for 5 min and quantitated as freezing time.

To examine the effects of i.n. CGRP on fear memory extinction, mice were fear conditioned in a chamber using foot shocks. The following day (Day 2), mice were placed in the same chamber. We measured mice freezing behavior for 30 min and then administered CGRP or saline by nasal treatment. On the following day (Day 3), freezing time was observed for 5 min.

### Analysis of CGRP contents

Mice were anesthetized with a mixture of three anesthetic agents (medetomidine hydrochloride 0.3 mg/kg, midazolam 4.0 mg/kg, and butorphanol 5.0 mg/kg) administered intraperitoneally as previously described^3^. Mice serum, CSF, or hippocampus samples were collected 30 min after i.n. CGRP administration. CGRP levels were measured using a sandwich ELISA kit (Bertin Technologies, Montigny-le-Bretonneux, France).

### Quantitative analysis by real-time PCR

Real-time PCR was carried out as previously described^3^. The sequences of primers, designed by the authors, were based on the coding sequences of mouse genes deposited in GenBank. The data were analyzed using the mean threshold cycle equation. The primer information is shown in Table 1.

**Table 1:** Oligonucleotide sequences for real-time PCR amplification.

	Forward	Reverse
Npas4	5′-CATGCTAAGGACCTAGCCCTACTG-3'	5′-GGTGTAGCAGTCCATACCATGA-3′
Actin	5′-GGTCAGAAGGACTCCTATGTG-3′	5′-GGTGTGGTGCCAGATCTTCTCC-3′

### Western blotting

We conducted western blot analyses as previously described^3^. Briefly, the collected hippocampus was homogenized in a sodium dodecyl sulfate (SDS) sample buffer and boiled for five minutes. Protein extracts were separated by SDS–polyacrylamide gel electrophoresis and then transferred onto a polyvinylidene difluoride membrane (HybondP; GE Healthcare UK Ltd.). The membrane was blocked with a blocking agent (GE Healthcare) and then incubated at 4 °C overnight with the following primary antibodies: mouse monoclonal anti-Npas4 (1:5,000, Santa Cruz Biotechnology, Inc.), rabbit polyclonal anti-HDAC5 (phospho S498) (1:5,000, Abcam plc, Cambridge, UK), mouse monoclonal anti-HDAC5 (1:5,000, Santa Cruz Biotechnology), rabbit polyclonal anti-PKD1/2/3 PKC micro antibody (Gene Tex, Inc. CA), mouse anti-beta actin antibody (1:20,000; Sigma-Aldrich) and rabbit anti-GAPDH antibody (1:20,000; Sigma-Aldrich CO. LLC. Japan). After washing, the membranes were incubated with horseradish peroxidase-conjugated secondary antibody (1:20,000). The antibody-reactive bands were visualized using a chemiluminescent substrate kit (FUJIFILM Wako Chemicals, Tokyo, Japan).

### Statistical analysis

GraphPad Prism 9 software (GraphPad Software Inc., San Diego, CA, USA) was used for main statistical analyses. G*Power 3 (Faul, Erdfelder, Lang & Buchner, 2007: http://www.psycho.uni-duesseldorf.de/abteilungen/aap/gpower3/) was used for power analyses. The results of power analysis showed in Supplementary 1: Data. Data analysis of mice was performed blind to group assignment. All data are expressed as the mean ± S.E.M. Comparisons between two values were analyzed using the Welch’s *t* test. We also performed Tukey–Kramer multiple comparison test to determine significant differences. We used the ROUT test to remove outliers from groups. A P value < 0.05 was considered statistically significant.

## Supplementary Information

Supplementary Information 1.

Supplementary Information 2.
