# Supplementary material for: Intranasal calcitonin gene-related peptide administration impairs fear memory retention in mice through the PKD/p-HDAC5/Npas4 pathway
Source: Sci Rep. 2022 Jan 27;12:1450. doi: 10.1038/s41598-022-05518-y (PMC8795377; doi:10.1038/s41598-022-05518-y)
Supplement: Supplementary file 2 — Supplementary Information 2. [file 41598_2022_5518_MOESM2_ESM.docx]

**Title:** Intranasal calcitonin gene-related peptide administration impairs fear memory retention in mice through the PKD/p-HDAC5/Npas4 pathway

Authors: Narumi Hashikawa-Hobara^1*^, Yoshikazu Yoneyama^1^, Kyoushiro Fujiwara^1^, Naoya Hashikawa^1^

**Affiliations:**

^1^ Department of Life Science, Okayama University of Science, 1-1 Ridai-cho, Kita-ku, Okayama 700-0005, Japan.

*To whom correspondence should be addressed: Narumi Hashikawa-Hobara, 1 Ph.D.

^1^Department of Life Science, Okayama University of Science, 1-1 Ridai-cho, Kita-ku Okayama 700-0005, Japan

+81-86-256-9719

E-mail: [hobara@dls.ous.ac.jp](mailto:hobara@dls.ous.ac.jp)

**Supplementary Data**

Fig. S1

Supplementary Figure 1,

Fig. S1. Anesthesia did not affect CGRP-mediated freezing percentage. After electrical foot shock, mice were administered saline or CGRP under anesthesia. Each bar indicates the mean ± S.E.M. * p < 0.05. Numbers in parentheses indicate the animal numbers for each group.

Supplementary Figure 2

Figure S2. The full-length blots of Figure 3A, 3B, 3D and 4B are presented.
